# Supplementary material for: Clicker Training Mice for Improved Compliance in the Catwalk Test
Source: Animals (Basel). 2022 Dec 15;12(24):3545. doi: 10.3390/ani12243545 (PMC9774362; doi:10.3390/ani12243545)
Supplement: Supplementary file 1 [file animals-12-03545-s001.zip › animals-2079910-supplementary.pdf]

Supplementary information

Article

Clicker training mice for improved compliance in the Catwalk test

Jana Dickmann[1], Fernando Gonzalez-Uarquin[1], Sandra Reichel[1], Dorothea Pichl[1], Konstantin Radyushkin[1], Jan Baumgart[1] and Nadine Baumgart[1*]

[1] Translational Animal Research Center, University Medical Center of the Johannes Gutenberg-University, Mainz, Germany
* Correspondence: nadine.baumgart@uni-mainz.de

1. Results

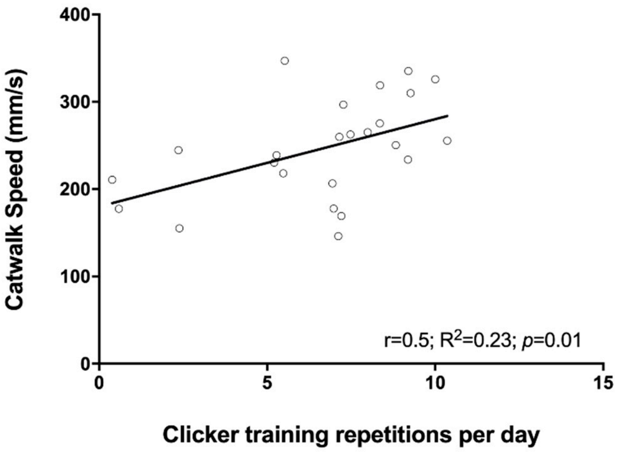

Supplementary Figure S1. Pearson's correlation test between clicker training (repetitions per day) and running speed (mm/s) in the CatWalk test. Each point represents an individual.

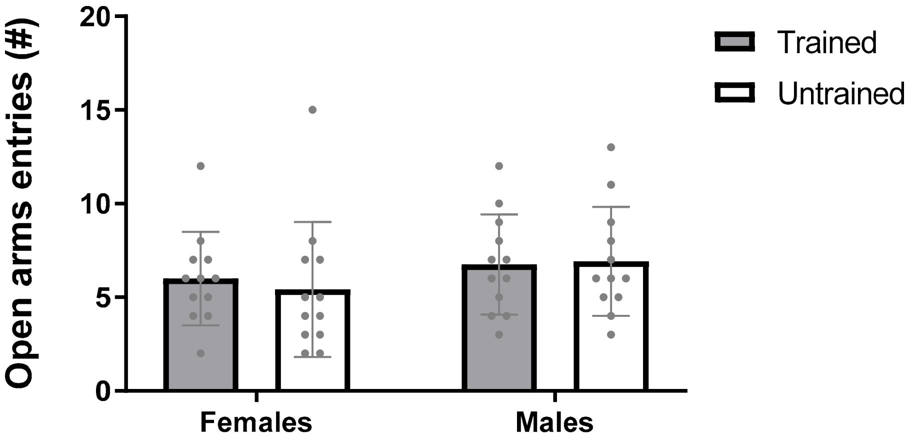

Supplementary Figure S2. Number of entries in the open arms of the Elevated Plus Maze (EPM) of previously trained (grey) and untrained (white) C57BL/6J mice. We used Two-way ANOVA for statistical analysis. n = 12. Each point represents an individual. Bars indicate the means ± standard deviation.
